# Analytical Model for Current–Voltage Characteristics in Perovskite Solar Cells Incorporating Bulk and Surface Recombination

**DOI:** 10.3390/mi15080972

**Published:** 2024-07-29

**Authors:** M. Z. Kabir

**Affiliations:** Department of Electrical and Computer Engineering, Concordia University, 1455 Boul. de Maisonneuve Ouest, Montreal, QC H3G 1M8, Canada; zahangir.kabir@concordia.ca

**Keywords:** perovskite solar cells, current–voltage characteristics, bulk and surface recombination, surface recombination velocity

## Abstract

The effects of surface recombination on the steady-state carrier profiles and photocurrent in perovskite solar cells are investigated in this paper. The continuity equations for both holes and electrons are solved considering carrier drift and diffusion under the exponential carrier generation profile in the perovskite layer and considering both bulk and interface carrier recombination. An analytical expression for the solar-induced photocurrent is derived. The rate of carrier recombination at the interfaces has a very significant effect on the carrier profile, photocurrent, and, hence, on the charge collection efficiency. The external current density is calculated considering the dark current and nominal solar spectrum-induced photocurrent. The proposed model is fitted and verified with published experimental results from various publications. The fittings of the model with experimental results provide information about the interface and bulk charge carrier transport parameters.

## 1. Introduction

The halide perovskite solar cells (PSCs) have received huge attention in recent years [1,2] due to their higher power conversion efficiency (PCE) and cheaper manufacturing cost. The PCE of PSCs has exceeded 25% in single-junction cells, which is comparable to highly researched silicon solar cells [3,4]. The perovskite materials possess several intrinsic qualities, such as a high absorption coefficient over the entire sun spectra and good charge carrier transport properties.

The general form of the halide perovskite materials is ABX3 (where A = CH3NH3 (abbreviated as MA+), HC(NH_2_)_2_ (abbreviated as FA+), and Cs; B = Pb^2+^ or Sn^2+^; and X is a halogen atom such as Cl^−^, I^−^, or Br^−^). The halide perovskites, such as MAPbI_3_, MAPbI_3−x_Cl_x_, and (FAPbI_3_)_x_(MAPbBr_3_)_1−x_, have been examined for photovoltaic solar cell applications. The bandgap of these perovskites varies within 1.5–1.6 eV [5,6], which is appropriate for photovoltaic solar cells. Both crystalline and polycrystalline forms of these perovskites can be used.

The perovskite photovoltaic solar cells fall within the thin-film photovoltaic structure category. The general structure is transparent electrode/HTL/bulk perovoskite/ETL/electrode type (where HTL and ETL stand for hole transport layer and electron transport layer, respectively). The top contact is made of transparent conductive oxide, and the bottom contact can be a metal. The bulk perovskite layer is an undoped (intrinsic type or slightly *n* or *p* type) layer, and its thickness is less than 1 μm [7]. The ETL and HTL are much thinner than the bulk perovskite layer. The structure is equivalent to ETL-i-HTL or HTL-i-ETL. The conventional structure is the ETL-i-HTL type, which provides higher efficiency. On the other hand, the HTL-i-ETL structure provides better stability and lower cost, with lower efficiency. Though the ETL and HTL are not conventional *n* and *p* layers, the carrier transport properties of these layers are like *n* and *p* layers. This means that the electron transport is much more favorable than the hole transport in ETL, and the opposite is true in HTL. The energy band of the ETL and HTL must align with the perovskite layer so that photogenerated electrons and holes can easily pass towards the corresponding electrodes. The ETL and HTL also play a very crucial role in the interface properties of the perovskite layer and, thus, the control carrier profile across the perovskite layer and the photocurrent as well. It is very crucial to investigate the quantitative effects of the interfaces on the current–voltage (J–V) characteristics of these photovoltaic cells.

There are a limited number of papers found in the literature that describe a physics-based model for calculating the photocurrent in perovskite solar cells [8,9,10]. These publications did not consider the combined effects of several main charge carrier transport mechanisms, such as carrier diffusion, bulk, and surface recombination, for calculating the photocurrent. Many previous publications [11,12,13,14] experimentally demonstrated that the PCE highly depends on the transport and contact properties of the ETL and/or HTL layers by changing the composition of these ETL and HTL layers. However, as per my knowledge, only one paper in the literature [15] proposed a physics-based analytical model for studying the current–voltage characteristics in perovskite solar cells incorporating a few important charge carrier transport mechanisms, such as carrier diffusion, bulk, and surface recombination. However, the mathematical derivation of the previous model was based on a few unrealistic assumptions, such as the same surface recombination velocity for both electrons and holes at both interfaces and the same carrier transport properties for both holes and electrons.

In this paper, the previous model [15] has been extended by incorporating unequal values of surface recombination velocity for electrons and holes. The effects of surface recombination on steady-state electron and hole profiles are examined by solving the carrier continuity equation across the perovskite layer and by varying the surface recombination velocities at both interfaces. The theoretical model is compared with published experimental results, and hence, the physical mechanisms for the variation in the charge collection efficiency with the change in ETL and HTL are investigated. The charge carrier transport properties are also extracted.

## 2. Mathematical Model

The sun excitation creates an exponential distribution of electron–hole pairs (EHPs) across the bulk perovskite layer, and thus, the carrier generation rate *G* is the following [16]:

(1) $$G(\lambda ,x)=G_{0}(\lambda )e^{-\alpha (\lambda )x}$$

and

(2) $$G_{0}\left(\lambda ,0\right)=\alpha \left(\lambda \right)\left[1-R\left(\lambda \right)\right]I_{0}\left(\lambda \right)/E_{ph}$$

where *E_ph_* = *hc*/*λ* is the incident photon energy, *h* is the Plank constant, *c* is the speed of light, *λ* is the wavelength of the incident photon, *x* is the distance from the radiation-receiving electrode, *R* is the refection loss from the front surface, *G*_0_(*λ*) is the EHP carrier generation rate at *x* = 0, *α*(*λ*) is the absorption coefficient of the perovskite material, and *I*_0_ is the intensity of the sun light.

The sunlight generated electrons and holes drift under the built-in electric field. The carriers may also move by the diffusion process. Some of the carriers are lost during their travel towards the charge-collecting electrodes. The monomolecular recombination is the dominant carrier loss mechanism because of the low bimolecular recombination coefficient in perovskites and relatively low light intensity [17]. Since our aim is to develop a compact analytical model, we make a few assumptions, such as [18] (1) the electric field *F* is approximately uniform [19] across the relatively thin perovskite layer under normal sun exposure, (2) the drift mobility (*μ*) and carrier lifetime (*τ*) in the active layer for each carrier type (electrons and holes) are uniform, and (3) the intrinsic carrier concentration for the relatively large bandgap (~1.5 eV) perovskites is negligible compared to the photogenerated carrier concentration.

The excess carrier continuity equations for holes and electrons in the bulk of the ETL-i-HTL structure under steady-state condition are the following:

(3) $$D_{p}\frac{\partial^{2}\delta_{p}}{\partial x^{2}}-\mu_{p}F\frac{\partial \delta_{p}}{\partial x}+Ge^{-\alpha (\lambda )x}-\frac{\delta_{p}}{\tau_{p}}=0$$

(4) $$D_{n}\frac{\partial^{2}\delta_{n}}{\partial x^{2}}+\mu_{n}F\frac{\partial \delta_{n}}{\partial x}+Ge^{-\alpha (\lambda )x}-\frac{\delta_{n}}{\tau_{n}}=0$$

where *δ* and *D* are the photogenerated excess carrier concentration and diffusion coefficient, respectively, while the subscripts *n* and *p* represent electrons and holes, respectively.

As mentioned previously, the ETL and HTL are not conventional *n* and *p* layers of homogeneous material. The electron concentration at the top surface (*x* = 0) of the perovskite layer may not be equal to the electron concentration of the ETL; rather, it should be determined by solving the recombination current density at the surface. The same argument applies to the hole concentration at the bottom interface (*x* = *W*). Therefore, all the boundary conditions for the carrier concentrations are determined by the surface recombination current. The boundary values of hole and electron current densities are the following [9,20,21]:

(5a) $$J_{p}\left(0\right)=q\mu_{p}F\delta_{p}(0)-qD_{p}\frac{\partial \delta_{p}(0)}{\partial x}=-qS_{pt}\delta_{p}(0)$$

(5b) $$J_{p}\left(W\right)=q\mu_{p}F\delta_{p}(W)-qD_{p}\frac{\partial \delta_{p}(W)}{\partial x}=qS_{pb}\delta_{p}(W)$$

(5c) $$J_{n}\left(0\right)=q\mu_{n}F\delta_{n}(0)+qD_{n}\frac{\partial \delta_{n}(0)}{\partial x}=qS_{nt}\delta_{n}(0)$$

(5d) $$J_{n}\left(W\right)=q\mu_{n}F\delta_{n}(W)+qD_{n}\frac{\partial \delta_{n}(W)}{\partial x}=-qS_{nb}\delta_{n}(W)$$

where *q* is the elementary charge, *W* is the thickness of the perovskite layer, *J* is the conduction current density, and *S* is the surface recombination velocity. The first subscripts *n* and *p* on *S* represent electrons and holes, while the second subscripts *t* and *b* represent the top and bottom interfaces, respectively. Considering the boundary conditions in Equation (5) and solving Equations (3) and (4), the excess carrier concentrations are the following:

(6) $$\delta_{p}(x,\lambda )=C_{1}e^{m_{1}x}+C_{2}e^{m_{2}x}+A_{1}e^{-\alpha x}$$

(7) $$\delta_{n}(x,\lambda )=C_{3}e^{m_{3}x}+C_{4}e^{m_{4}x}+A_{2}e^{-\alpha x}$$

where

$$\begin{array}{c} m_{1}=\frac{\mu_{p}F+\sqrt{{\mu_{p}}^{2}F^{2}+4\frac{D_{p}}{\tau_{p}}}}{2D_{p}},\;m_{2}=\frac{\mu_{p}F-\sqrt{{\mu_{p}}^{2}F^{2}+4\frac{D_{p}}{\tau_{p}}}}{2D_{p}}{,\;A}_{1}=\frac{G\tau_{p}}{1-\mu_{p}F\alpha \tau_{p}-D_{p}\alpha^{2}\tau_{p}},\\ m_{3}=\frac{-\mu_{n}F+\sqrt{{\mu_{n}}^{2}F^{2}+4\frac{D_{n}}{\tau_{n}}}}{2D_{n}},\;m_{4}=\frac{-\mu_{n}F-\sqrt{{\mu_{n}}^{2}F^{2}+4\frac{D_{n}}{\tau_{n}}}}{2D_{n}}{\;,\;A}_{2}=\frac{G\tau_{n}}{1+\mu_{n}F\alpha \tau_{n}-D_{n}\alpha^{2}\tau_{n}},\\ C_{1}=\left(A_{1}/K_{1}\right)\left[e^{-\alpha W}\left(D_{p}m_{2}-\mu_{p}F-S_{pt}\right)\left(\alpha D_{p}+\mu_{p}F-S_{pb}\right)-e^{m_{2}W}\left(D_{p}m_{2}-\mu_{p}F+S_{pb}\right)\left(\alpha D_{p}+\mu_{p}F+S_{pt}\right)\right],\;\\ C_{2}=\left(A_{1}/K_{1}\right)\left[e^{m_{1}W}\left(D_{p}m_{1}-\mu_{p}F+S_{pb}\right)\left(\alpha D_{p}+\mu_{p}F+S_{pt}\right)-e^{-\alpha W}\left(D_{p}m_{1}-\mu_{p}F-S_{pt}\right)\left(\alpha D_{p}+\mu_{p}F-S_{pb}\right)\right],\;\\ C_{3}=\left(A_{2}/K_{2}\right)\left[e^{-\alpha W}\left(-D_{n}m_{4}-\mu_{n}F+S_{nt}\right)\left(-\alpha D_{n}+\mu_{n}F+S_{nb}\right)-e^{m_{4}W}\left(-D_{n}m_{4}-\mu_{n}F-S_{nb}\right)\left(-\alpha D_{n}+\mu_{n}F-S_{nt}\right)\right],\;\\ C_{4}=\left(A_{2}/K_{2}\right)\left[e^{m_{3}W}\left(-D_{n}m_{3}-\mu_{n}F-S_{nb}\right)\left(-\alpha D_{n}+\mu_{n}F-S_{nt}\right)-e^{-\alpha W}\left(-D_{n}m_{3}-\mu_{n}F+S_{nt}\right)\left(-\alpha D_{n}+\mu_{n}F+S_{nb}\right)\right],\;\\ K_{1}=e^{m_{1}W}\left(D_{p}m_{2}-\mu_{p}F-S_{pt}\right)\left(D_{p}m_{1}-\mu_{p}F+S_{pb}\right)-e^{m_{2}W}\left(D_{p}m_{1}-\mu_{p}F-S_{pt}\right)\left(D_{p}m_{2}-\mu_{p}F+S_{pb}\right),\;\mathrm{and}\\ K_{2}=e^{m_{3}W}\left(-D_{n}m_{4}-\mu_{n}F+S_{nt}\right)\left(-D_{n}m_{3}-\mu_{n}F-S_{nb}\right)-e^{m_{4}W}\left(-D_{n}m_{3}-\mu_{n}F+S_{nt}\right)\left(-D_{n}m_{4}-\mu_{n}F-S_{nb}\right). \end{array}$$

The photocurrent density for the incident photon of wavelength *λ* can be conveniently determined using the Shockley–Ramo theorem [22,23], which is given by the following:

(8) $$\begin{array}{cc} J_{ph}(\lambda ,V) & =\frac{q}{W}[\{\mu_{p}F\int_{0}^{W}\delta_{p}\partial x-D_{p}\int_{0}^{W}\frac{\partial \delta_{p}}{\partial x}\partial x\}+\{\mu_{n}F\int_{0}^{W}\delta_{n}\partial x+D_{n}\int_{0}^{W}\frac{\partial \delta_{n}}{\partial x}\partial x\}]\\ & =\frac{q}{W}[\mu_{p}F\{\frac{C_{1}}{m_{1}}(e^{m_{1}W}-1)+\frac{C_{2}}{m_{2}}(e^{m_{2}W}-1)-\frac{A_{1}}{\alpha }(e^{-\alpha W}-1)\}\\ & -D_{p}\{C_{1}(e^{m_{1}W}-1)+C_{2}(e^{m_{2}W}-1)+A_{1}(e^{-\alpha W}-1)\}\\ & +\mu_{n}F\{\frac{C_{3}}{m_{3}}(e^{m_{3}W}-1)+\frac{C_{4}}{m_{4}}(e^{m_{4}W}-1)-\frac{A_{2}}{\alpha }(e^{-\alpha W}-1)\}\\ & +D_{n}\{C_{3}(e^{m_{3}W}-1)+C_{4}(e^{m_{4}W}-1)+A_{2}(e^{-\alpha W}-1)\}]\; \end{array}$$

The entire sun spectra contribute to the photocurrent and, thus, the total photocurrent density:

(9) $$J_{ph}(V)=\int_{0}^{\infty }J_{ph}(\lambda ,V)d\lambda$$

Most of the external voltage drops can occur across the perovskite layer because the very thin ETL and HTL are more conductive than the perovskite layer. Thus, the expression of the electric field *F* is the following:

(10) $$F\approx \frac{V_{bi}-(V-JR_{s})}{W}$$

where *V_bi_* is the built-in potential, *R_s_* is the effective series area resistance, and *V* and *J* are the external load voltage and current density, respectively.

The external current density from a solar cell is given by the following [24,25]:

(11) $$J(V)=J_{d}(V)+\frac{V-R_{s}J(V)}{R_{p}}-J_{ph}(V),$$

where *J_d_*(*V*) and *R_p_* are the dark current density and the shunt area resistance, respectively. The expression of the dark current density is given by the following [15,26]:

(12) $$J_{d}=J_{c}\frac{V_{t}}{V_{bi}-(V-JR_{s})}\times \{\exp(\frac{V-JR_{s}}{2V_{t}})-\exp[\frac{2(V-JR_{s})-V_{bi}}{2V_{t}}]\},$$

where *V_t_* = *k_b_T*/*q*, *T* is the absolute temperature, and *k_b_* is the Boltzmann constant. The expression of dark current (Equation (12)) has a voltage-independent constant term *J_c_*, which is considered as a fitting parameter.

## 3. Results and Discussion

The Equations (8) to (12) are simultaneously solved to find the net current density *J* at a particular external voltage *V*. The air mass (AM) 1.5 global spectra are used as the irradiation to the solar cells [27]. The mobility and lifetime of carriers vary widely in perovskite materials depending on the material compositions and deposition conditions [28]. The electron and hole mobilities in crystalline perovskites vary from 10 to more than 100 cm^2^/Vs, whereas in polycrystalline perovskites, these are ~1–10 and ~5–20 cm^2^/Vs, respectively [29,30]. The carrier lifetime usually varies in the range 0.1–100 µs in perovskite films [7].

### 3.1. Effects of Surface Recombination

**Figure 1:**
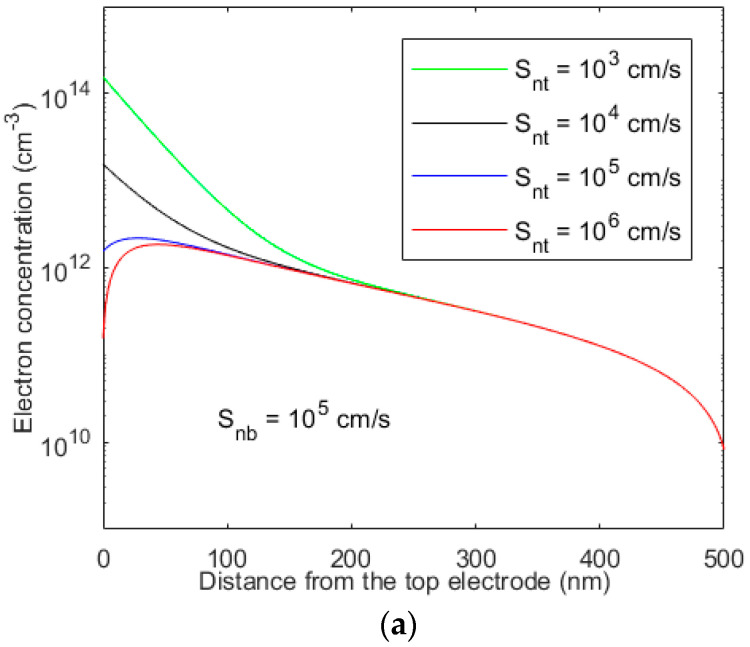

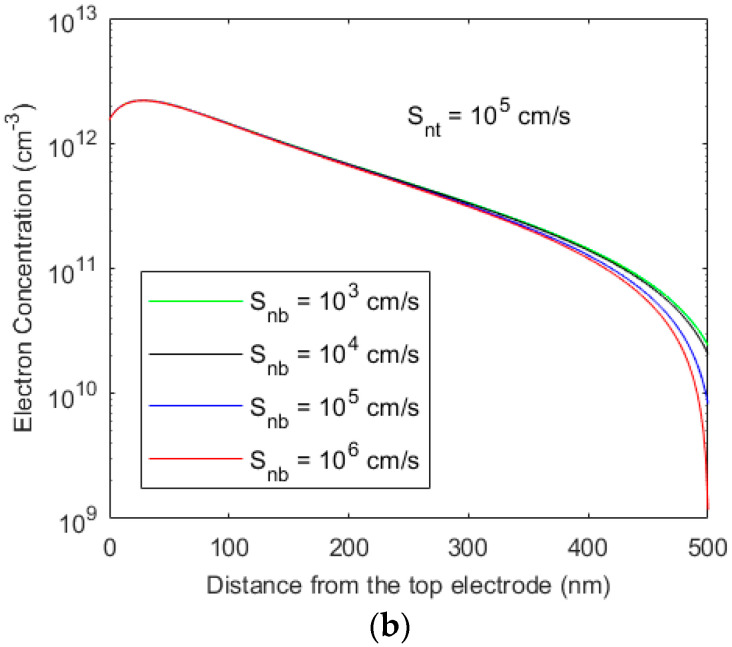
The profile of photogenerated electron concentration across the perovskite layer in an ETL-i-HTL type structure for various recombination velocities at both ends.

**Figure 2:**
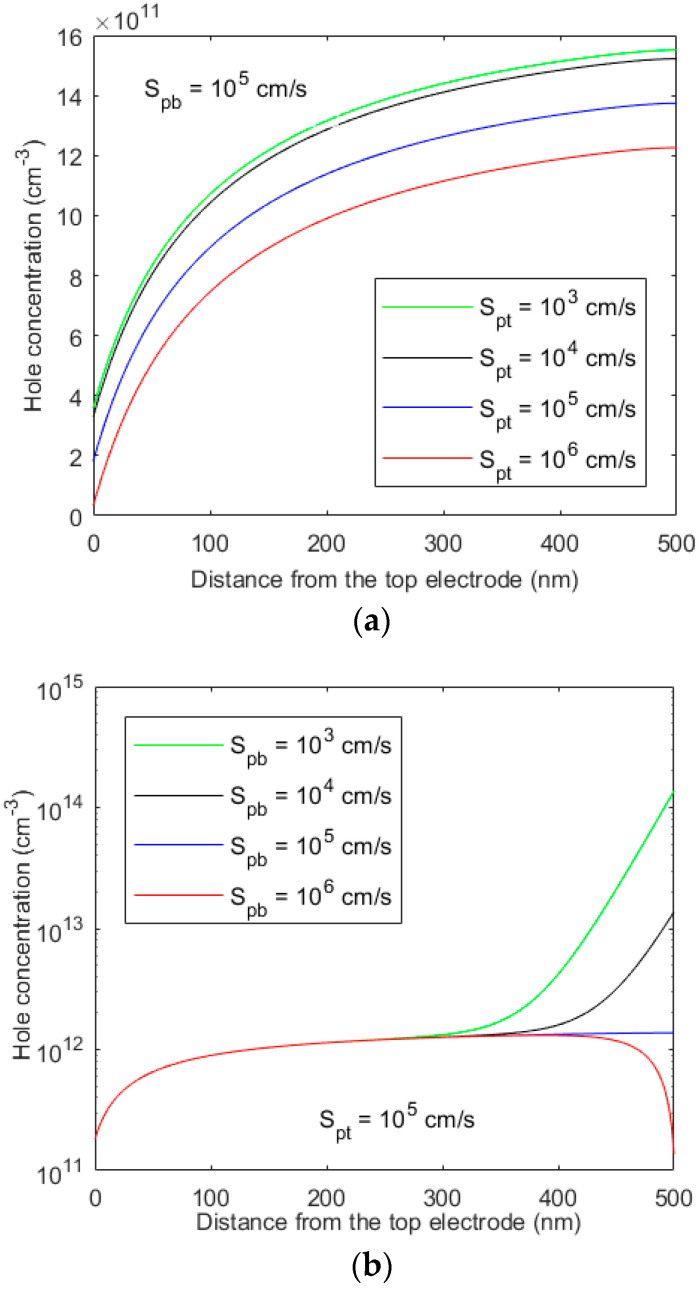
The profile of photogenerated hole concentration across the perovskite layer in an ETL-i-HTL type structure for various recombination velocities at both ends.

Figure 1 and Figure 2 show the photogenerated electron concentration profiles across a 0.5 µm-thick perovskite layer in an ETL-i-HTL type structure as a function of surface recombination velocities at both ends. The following parameters are assumed [15]: *V_bi_* = 1.2 V, *µ_p_* = 10 cm^2^ V^−1^ s^−1^, *µ_n_* = 5 cm^2^ V^−1^ s^−1^, *τ_n_* = *τ_p_* = 10 μs, and *F* = 1 V/μm. The absorption depth (i.e., 1/*α*) for the entire sun spectra is smaller than 0.2 μm. As shown in Figure 1 and Figure 2, the carrier profile is more dependent on the surface recombination velocity of (Figure 2a) the carrier type that moves towards the bottom electrode (holes in this case). Again, the surface recombination velocity at the top contact for this carrier type critically controls the carrier profile (Figure 1a) and, thus, the overall photocurrent as well. In general, the photogenerated carrier concentration decreases with an increase in surface recombination velocity. The surface recombination velocity increases with increasing trapped carrier concentration at the interface between the perovskite and ETL or HTL layer. The interface properties between the ETL and perovskite have a more significant effect on the carrier profile.

**Figure 3:**
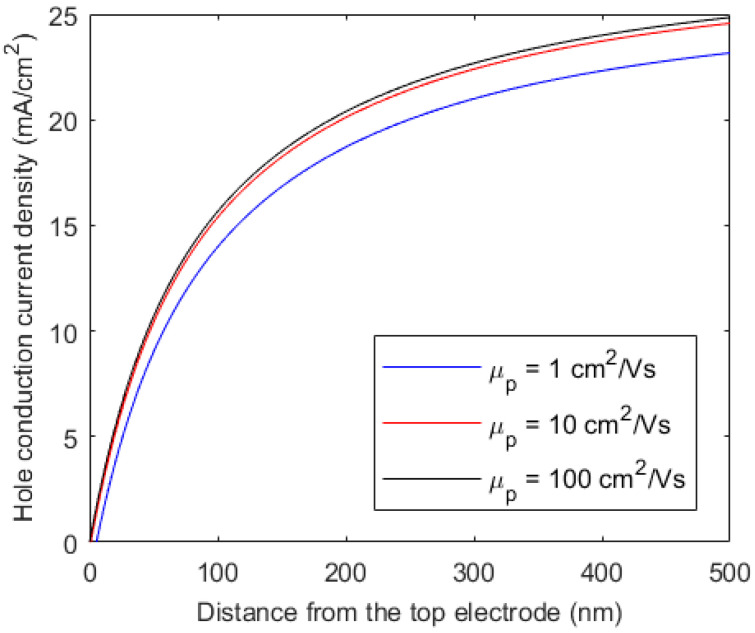
Hole conduction current densities versus distance from the radiation-receiving electrode for different hole mobilities. Hole lifetime, *τ_p_* = 10 μs.

Among four transport parameters (i.e, drift mobilities and carrier lifetimes of holes and electrons), the drift mobility for the carriers that drift towards the back electrode has the most significant effect on the overall charge collection efficiency. Considering that holes are drifting towards the bottom electrode, the effect of hole drift mobility on the hole conduction current density at *V* = 0 is shown in Figure 3. The surface recombination velocities are *S_pb_* = *S_pt_* = *S_nt_* = *S_nb_* = 10^4^ cms^−1^. The effects of other transport parameters of the bulk (i.e., electron drift mobility, electron lifetime, and hole lifetime) are almost negligible for this very thin (less than 1 μm) perovskite layer. Note that the effects of the hole and electron transport properties will be interchanged in the HTL-i-ETL type structure.

**Figure 4:**
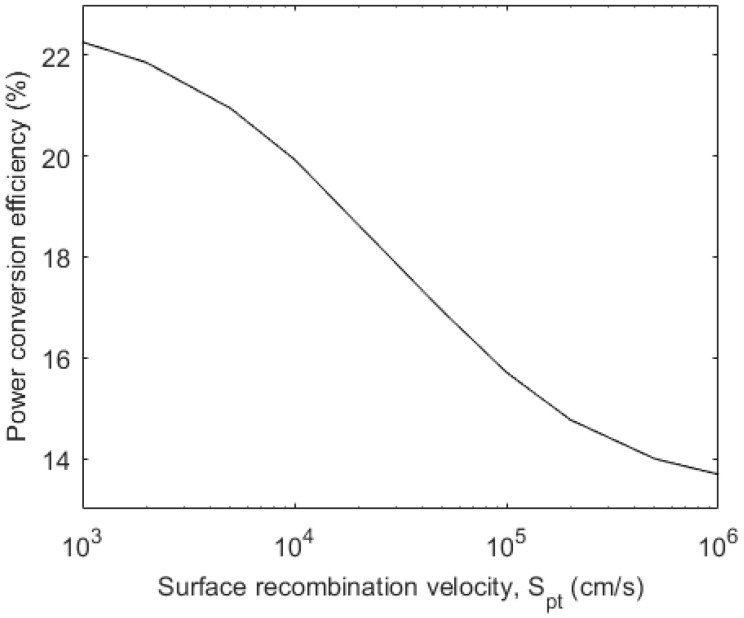
PCE versus *S_pt_* of a MAPBI_3_-based ETL-i-HTL type solar cell.

As mentioned earlier, the surface recombination velocity at the top contact for the carriers that drift towards the back electrode critically controls the carrier profile and, hence, the overall photocurrent. Therefore, it is instructive to show this effect on the overall conversion efficiency. Figure 4 shows the effect of the surface recombination velocity at the top surface (*S_pt_*) on the overall PCE of the same cell as in Figure 1 and Figure 2. The other surface recombination velocities are *S_pb_* = *S_nt_* = *S_nb_* = 2 × 10^4^ cms^−1^. The carrier transport parameters in Figure 4 are the same as in Figure 1, with *R_s_* = 3 Ωcm^2^, *R_p_* = 10^4^ Ωcm^2^, and *J_c_* = 3 × 10^−9^ mAcm^−2^. As shown in Figure 4, the surface recombination velocity at the top surface (*S_pt_*) has a strong effect on the PCE.

### 3.2. Model Fitting with Measurement Data

**Figure 5:**
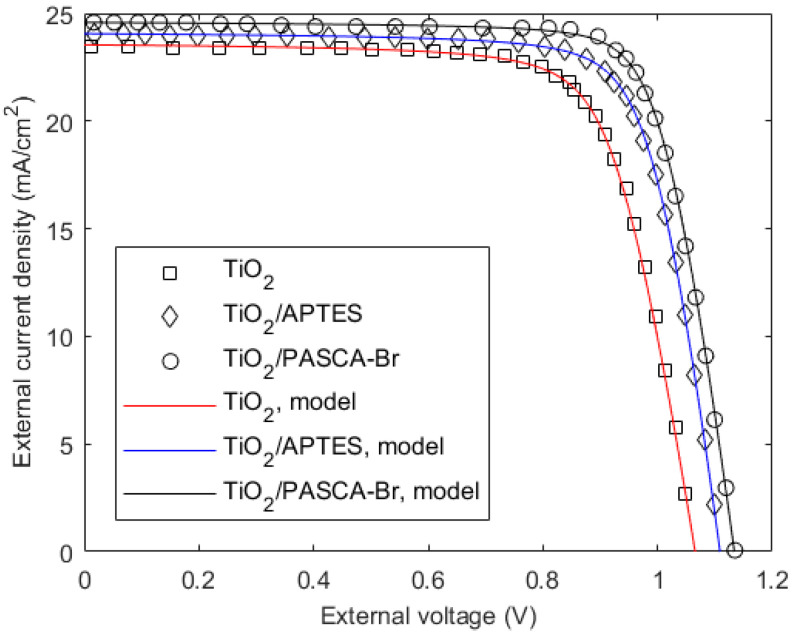
Current−voltage characteristics of a Cs_0.05_(FA_0.83_MA_0.17_)_0.95_Pb(I_0.83_ Br_0.17_)_3_-based solar cell on FTO substrate. Symbols represent extracted experimental data from Figure 4 of [14]. The legends represent compositions of the ETL layer.

The theoretical model fitting with the experimental results of a Cs_0.05_(FA_0.83_MA_0.17_)_0.95_Pb(I_0.83_ Br_0.17_)_3_-based solar cell on the FTO substrate is shown in Figure 5. The material composition of the structure is FTO/TiO_2_(TiO_2_/APTES/PASCA-Br)/Cs_0.05_(FA_0.83_MA_0.17_)_0.95_Pb(I_0.83_ Br_0.17_)_3_/PCBM/Ag [14], which is the ETL-i-HTL type. The perovskite layer thickness is 0.4 μm. The symbols and solid lines represent the experimental data and the model fittings to the experimental results, respectively. The three structures have different interfacial materials at the ETL/perovskite interface. The transport properties of electrons and holes are not equal in perovskite materials. Since these values are not precisely known, they are assumed equal in this experimental fitting in order to reduce the fitting parameters. The mathematical model considers separate values for electron and hole transport parameters, and thus, one can easily change these values in the calculation if these values are known. The transport parameters are assumed as *µ_p_* = 10 cm^2^ V^−1^ s^−1^, *µ_n_* = 5 cm^2^ V^−1^ s^−1^, and *τ_n_* = *τ_p_* = 1 μs, which are typical values for perovskites. Since the charge collection efficiency mostly depends on the carriers that move towards the bottom electrode (holes in this case), the assumed values are accurate for this carrier type. The transport parameters for other types of carriers have little effect on the overall performance, and these mentioned values may not be very accurate. Again, to reduce the fitting parameters, the surface recombination velocity for electrons is assumed to be the same as that for holes. The other fitting parameters are given in Table 1. The change in the ETL layer changes the interface properties of the ETL and perovskite layer and so does the surface recombination velocity, *S_pt_*. The reduction in *S_pt_* and *R_s_* improves the PCE.

**Figure 6:**
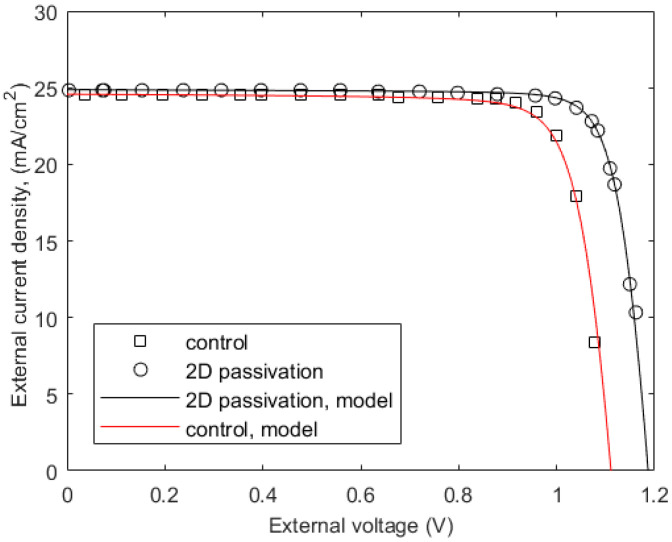
Current−voltage characteristics of a (FAPbI_3_)_0.95_(MAPbBr3)_0.05_-based solar cell. Symbols represent extracted experimental data from [31].

The theoretical fitting with the experimental J–V characteristics of a (FAPbI_3_)_0.95_(MAPbBr_3_)_0.05_-based solar cell is shown in Figure 6. The structure is the ETL-i-HTL type, and its material composition is FTO/SnO_2_/(FAPbI_3_)_0.95_(MAPbBr_3_)_0.05_/SpiroOMeTAD/Au. The symbols and solid lines represent the experimental data (extracted from Figure 16 of ref. [31]) and the model fittings to the experimental results, respectively. The structure mentioned above is the “control” structure, and the other structure has a 2D passivation of (BA)_2_PbI_4_ between the perovskite and HTL layer by the SIG processing method. The 2D passivation improves the interface properties and reduces the surface recombination velocity, as evident from Table 1, which leads to a higher open circuit voltage and better PCE.

## 4. Conclusions

A physics-based mathematical model for describing the current–voltage characteristics in perovskite solar cells has been developed by incorporating carrier drift, diffusion, and recombination in the bulk and interfaces. The individual effects of surface recombination at both ends on steady-state electron and hole profiles across the perovskite layer are studied. The theoretical calculations are compared with published experimental data and show excellent agreement. This theoretical model can serve as a tool for extracting charge carrier transport and cell parameters. The mathematical model can also be applied to other solar cell technologies if the cell structure is similar to PSC (for example, the structure of a bulk heterojunction organic solar cell is similar to PSC).

## Figures and Tables

**Table 1:** The extracted parameters for Figure 5 and Figure 6 are summarized in Table 1.

Bulk Perovskite	ETL/HTL Composition	*V_bi_* (V)	*J_c_* (Acm^−2^)	*R_s_* (Ωcm^2^)	*S_pt_* = *S_nt_* (cms^−1^)	*S_pb_* = *S_nb_* (cms^−1^)	PCE (%)
Cs_0.05_(FA_0.83_MA _0.17_)_0.95_Pb(I_0.83_ Br_0.17_)_3_ [14]	TIO_2_	1.08	1.5 × 10^−11^	4	5 × 10^3^	5 × 10^3^	18.3
	TIO_2_/APTES	1.13	1 × 10^−11^	3.2	4 × 10^3^	5 × 10^3^	20.3
	TIO_2_/PASCA-Br	1.15	0.5 × 10^−11^	3.2	2 × 10^3^	5 × 10^3^	21.6
(FAPbI_3_)_0.95_(MAPbBr_3_)_0.05_ [31]	Control (no 2D passivation)	1.22	4 × 10^−11^	1	4 × 10^2^	5 × 10^4^	22.5
	With 2D passivation	1.22	10^−12^	1	4 × 10^2^	5 × 10^2^	24.5

## Data Availability

The original contributions presented in the study are included in the article, further inquiries can be directed to the corresponding author.
